# Efficacy of bolus injections of landiolol hydrochloride as premedication in coronary artery CT angiography

**DOI:** 10.1186/s13244-024-01892-5

**Published:** 2025-01-10

**Authors:** Mark Barwig, Michael Janisch, Johannes Gessl, Wolfgang Kübler, Christopher König, Gerold Schwantzer, Helmut Schöllnast

**Affiliations:** 1 Institute of Radiology, LKH Graz II, Graz, Austria; 2 Division of Neuroradiology, Vascular and Interventional Radiology, Department of Radiology, Medical University of Graz, Graz, Austria; 3 Institute for Medical Informatics, Statistics and Documentation, Medical University of Graz, Graz, Austria; 4 Division of General Radiology, Department of Radiology, Medical University of Graz, Graz, Austria

**Keywords:** Multidetector computed tomography, Cardiac imaging techniques, Beta-adrenergic blocker, Heart–drug effects, Heart rate

## Abstract

**Purpose:**

To assess the efficacy of bolus injections of landiolol hydrochloride as premedication in coronary artery CT angiography (CCTA).

**Methods:**

The study population consisted of 37 patients (17 female; median age, 56 years; IQR, 19 years; range, 19–88 years) who underwent CCTA after intravenous injection of landiolol hydrochloride due to a heart rate > 60 bpm. Landiolol hydrochloride was administered in a stepwise manner until a heart rate of ≤ 60 bpm was achieved or a maximum dose of 60 mg was reached after six injections. Heart rates routinely displayed continuously on the CT scanner before the start of the landiolol hydrochloride injection (HR_PRE_), after each partial dose (HR_1–6_), during the CT scan (HR_CT_), and after the examination before moving from the CT table (HR_POST_) were recorded. Furthermore, the blood pressure routinely measured before (BP_PRE_) and after the examination before moving from the CT table (BP_POST_) was recorded.

**Results:**

A HR_CT_ of ≤ 60 bpm was achieved in 13 patients (35%) and a HR_CT_ ≤ 65 bpm was achieved in 25 patients (68%). The mean difference (± SD) between HR_PRE_ and HR_CT_ was −11 ± 9 bpm in total, −14 ± 10 bpm in patients without oral beta-blocker premedication and −6 ± 5 bpm in patients with oral Beta-blocker premedication.

**Conclusions:**

Landiolol hydrochloride enables a reduction of the heart rate in patients with and without oral beta-blocker premedication, whereby the use of serial partial doses is a simple and effective approach in clinical routine.

**Critical relevance statement:**

In cardiac CT, weight-independent, stepwise landiolol hydrochloride injection up to 40 mg reduces heart rate by −14 bpm without and −5 bpm with oral beta-blocker premedication, and achieves heart rates of ≤ 65 bpm in a significant proportion of patients.

**Key Points:**

- The ideal heart rate for cardiac CT is ≤ 60–65 bpm, which improves image quality and reduces radiation dose.
- In cardiac CT, landiolol hydrochloride intravenously reduces heart rate by −14 bpm.
- Heart rate of ≤ 65 bpm can be achieved in a significant proportion of patients.

**Graphical Abstract:**

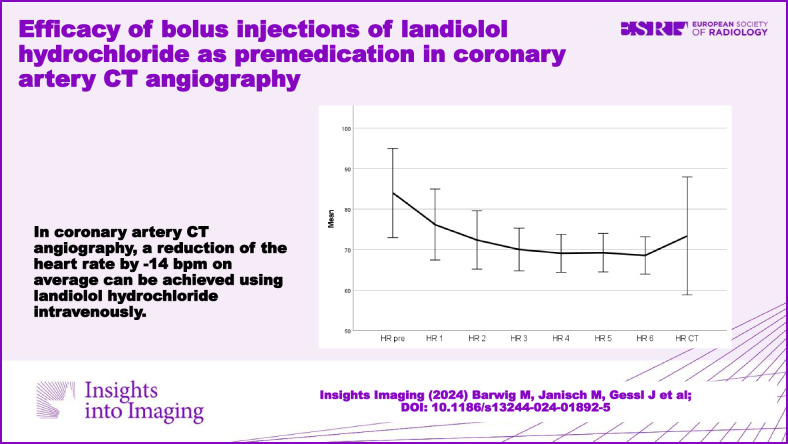

## Introduction

Coronary artery CT angiography (CCTA) is a non-invasive imaging modality for visualizing coronary artery stenosis in patients with known or suspected coronary artery disease. Depending on the technical properties of the CT scanner, high heart rate can produce motion artifacts that compromise image quality. Beta-blockers are usually used as premedication to lower the heart rate to a level suitable for CCTA. The goal is ideally a heart rate of ≤ 60–65 bpm, which not only improves image quality but also reduces radiation dose [1]. Both, oral and intravenous beta-blockers are used to achieve an optimal heart rate during the examination. Oral metoprolol is usually preferred as premedication, but it is most effective 1 h after administration. Intravenous beta-blockers are administered when on-site heart rate control is inadequate. Peak plasma levels of metoprolol following intravenous bolus injection are seen between 5 and 10 min. Elimination occurs in the liver with a mean plasma half-life of 3 h [2]. This long duration of action is suboptimal for use in CCTA because the examination itself lasts only a few minutes; thus, an efficacy of several hours far exceeds the necessary duration of action. Esmolol and landiolol are ultra-short-acting beta-blockers for intravenous administration only. Both substances are cardioselective with a very high β1-receptor selectivity. Compared to esmolol, landiolol has a much higher β1-receptor selectivity (landiolol: 216-fold β1-selectivity; esmolol: 30-fold β1-selectivity) [3]. Landiolol hydrochloride has an elimination half-life (t1/2) of about 4 min and is rapidly hydrolyzed in the blood and liver to the inactive metabolite and excreted in the urine [4]. Like all β1-adrenoreceptor antagonists, esmolol and landiolol have negative chronotropic and inotropic effects; the latter effect is less pronounced with landiolol [5, 6]. Because of these properties, landiolol hydrochloride seems to be more suitable than conventional beta-blockers in CCTA.

Landiolol hydrochloride is widely used in Japan (under the brand name Onoact), and since 2017, the European Union health authorities have approved usage under the name Rapibloc®. Landiolol was originally approved by Japan for treatment of intraoperative tachyarrhythmias and was later approved for tachycardia, atrial fibrillation, and atrial flutter during left ventricular dysfunction in November 2013. In 2019, landiolol expanded its utility into usage for fatal arrhythmia requiring emergency treatment and is becoming a reliable therapeutic choice for management of arrhythmia in acute phase [7].

To date, there are only a few studies on the efficacy of landiolol hydrochloride in the specific setting of frequency optimization for CCTA, and these studies have been mainly conducted in the Asian population [8–11]. Therefore, the aim of our study was to assess the efficacy of bolus injections of landiolol hydrochloride as premedication in CCTA in patients with and without oral beta-blocker premedication.

## Materials and methods

### Patient population

The ethics committee of the Medical University of Graz approved this study and informed consent was waived due to the retrospective study design.

**Fig. 1:**
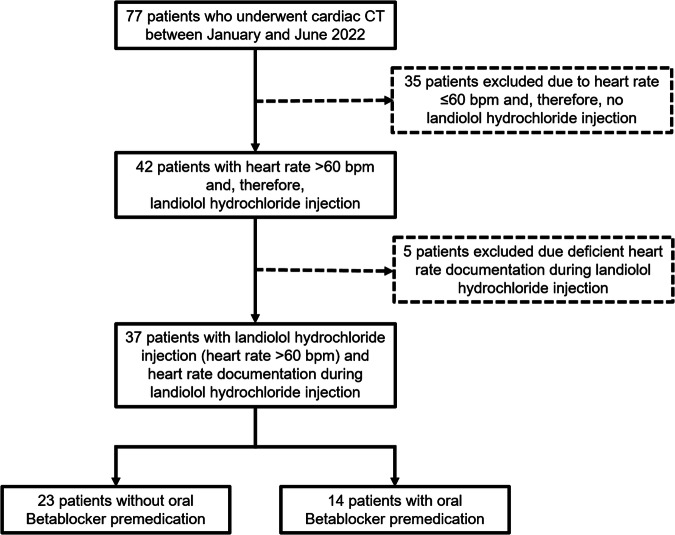
Flow chart showing patients’ inclusion and exclusion

A search of our institutional records yielded 77 patients who underwent CCTA between January and June 2022 at our institution in whom metoprolol was replaced by landiolol hydrochloride for intravenous premedication when undergoing the CCTA in January 2022 for clinical routine. Out of them, 42 patients received landiolol hydrochloride intravenously due to initial heart rate > 60 bpm. The remaining patients had a heart rate ≤ 60 bpm and did not receive landiolol hydrochloride intravenously. Prior to imaging, the medical history of all patients who were considered to receive landiolol hydrochloride was checked to rule out known contraindications for the administration of landiolol hydrochloride. All patients were older than 19 years of age, there were no pediatric CCTAs included in our study. In five patients, there was inadequate documentation of the heart rate during landiolol hydrochloride injection, and these patients were excluded. Thus, the final study population consisted of 37 patients who underwent CCTA after intravenous injection of landiolol hydrochloride due to a heart rate > 60 bpm. Figure 1 illustrates the inclusion and exclusion process in our study population.

### Landiolol hydrochloride administration

Landiolol hydrochloride was administered according to the manufacturer’s recommendations. Six partial injections (7 mg, 13 mg, 10 mg, 10 mg, 10 mg, and 10 mg) were administered in a stepwise manner (injection of each partial dose within 30 s with a gap of 15 s between each injection) with the patient on the CT table with observation of the heart rate until a heart rate of ≤ 60 bpm was achieved. A maximum of 60 mg of landiolol hydrochloride was administered, and CCTA was performed after this maximum dose was reached, regardless of the heart rate.

### Data acquisition

All examinations were performed on the same CT scanner (SOMATOM Definition Flash, Siemens Healthineers). CT without contrast for Calcium Scoring (collimation: 128 × 0.6 mm, pitch: 3.4, section thickness 3 mm, reconstruction interval 1.5 mm, Care dose 4D) was performed first followed by either helical CT (retrospective gating, *n* = 6), (collimation: 128 × 0.6 mm, pitch: 0.17, section thickness 0.6 mm, reconstruction interval 0.4 mm, Care dose 4D) or sequential CT (prospective triggering, *n* = 31), (collimation: 128 × 0.6 mm, section thickness 0.6 mm, reconstruction interval 0.4 mm, Care dose 4D). In patients with a body weight less than 80 kg, 70 mL Iopromid (Ultravist 370®, Bayer Vital GmbH) or Iomeprol (Iomeron 400®, Bracco) were injected following 10 mL of contrast as test bolus for bolus timing. In patients with a body weight of more than 80 kg, 90 mL of the same contrast media were injected. Contrast media injection was followed by 40 mL of saline chaser.

Heart rates routinely displayed continuously on the CT scanner were recorded before the start of the landiolol hydrochloride injection (HR_PRE_), after each partial dose (HR_1–6_), during the CT scan (HR_CT_), and after the examination before moving from the CT table (HR_POST_). Furthermore, the blood pressure routinely measured before (BP_PRE_) and after the examination before moving from the CT table (BP_POST_) was recorded.

### Statistical analysis

Continuous data are presented as medians with interquartile ranges (IQR) and ranges (Min., Max.). Categorical data are presented as frequencies and percentages. For the comparison of HR at different time points, the mean and median differences and standard deviations were calculated. The statistical significance of changes in HR and BP before and after landiolol hydrochloride injection and between the partial injections in patients who received the full dose (HR_1_–HR_6_) was tested using the Wilcoxon signed-rank Test. The significance of difference in change of HR between patients who received oral beta-blocker premedication and patients who did not, as well as between the sexes, was tested using the Mann–Whitney test. The correlation between HR change after landiolol hydrochloride injection and body weight, body height and body mass index was assessed using Spearman’s Rho. A *p* ≤ 0.05 was defined as statistically significant. SPSS Statistics 28 (IMB) was used for statistical evaluation.

## Results

The final study population consisted of 37 patients (20 male, 17 female). Median age was 56 years (IQR, 19 years; range, 19–88 years) who underwent CCTA after intravenous injection of landiolol hydrochloride due to a heart rate > 60 bpm. Median body weight was 77 kg (IQR, 32 kg; range, 50–130 kg), median body height was 172 cm (IQR, 12 cm; range, 150–190 cm), and median body mass index was 25 kg/m² (IQR, 7 kg/m²; range, 17–44 kg/m²). Fourteen patients received oral beta-blocker 1 h before the examination (Bisoprolol 1.25 mg in one patient, Bisoprolol 2.5 mg in five patients, Bisoprolol 5 mg in four patients, Bisoprolol 10 mg in one patient, Carvedilol 12.5 mg in one patient, Carvedilol 25 mg in one patient, and Nebivolol 2.5 mg in one patient), whereas in 23 patients no oral beta-blocker was administered by the referring physicians. 0.4 mg Glyceroltrinitrat was administered in 32 patients, in five patients glyceroltrinitrat was withhold due to lack of sufficient information on contraindication at the time point of examination at the Radiology.

Cardiac ultrasound was performed in 34/37 patients within 2 weeks to CCTA. Ejection fraction was normal in 29 patients (78.4%), moderately reduced in four patients (10.8%), and reduced in one patient (2.7%). In three patients (8.1%), no cardiac ultrasound within 2 weeks of CCTA was available.

All 37 individuals enrolled in the study received the study drug; however, only 15 patients received all six partial injections (a maximum amount of 60 mg) of landiolol hydrochloride. Given that the required heart rate (≤ 60 bpm) of the remaining 22 patients was received without using the maximum study dose of landiolol hydrochloride, the application of the remaining partial doses was relinquished. Three patients received one dose (7 mg), twelve patients received two doses (20 mg), no patient received three doses, seven patients received four doses (40 mg) and no patient received five doses. The mean dose (± SD) was 0.526 ± 0.3 mg/kg (95% CI, 0.426–0.626 mg/kg). No adverse effects occurred after landiolol hydrochloride administration in our patient cohort.

Image quality of the CCTA was categorized as excellent (no, to almost no motion artifacts) in 21 patients, good (minor motion artifacts without relevant influence on image interpretation) in 10 patients, moderate (some motion artifacts with little influence on image interpretation) in 5 patients, and poor (substantial motion artifacts with important influence on image interpretation) in 1 patient.

In 19 patients (51.4%), CCTA was reported as CAD-RADS 0, in 8 patients (21.6%) as CAD-RADS 1, in 1 patient (2.7%) as CAD-RADS 2, in 3 patients (8.1%) as CAD-RADS 3, in 4 patients (10.8%) as CAD-RADS 4a, and in 1 patient (2.7%) as CAD-RADS 4b. In 1 patient (2.7%), the CCTA study was non-diagnostic (CAD-RADS N).

Tables 1 and 2 show details on HR before landiolol hydrochloride injection, during using our scheme of fractional landiolol hydrochloride administration, during CCTA and after the CT examination as well as BP before and after CCTA. A statistically significant decrease of HR by −12 bpm during fractional landiolol hydrochloride administration compared to the initial HR was observed. We also found a statistically significant increase of HR by +2 bpm during CCTA compared to the lowest HR during fractional landiolol hydrochloride administration. Despite the increase of HR during CCTA compared to the HR during fractional landiolol hydrochloride administration, there was still a statistically significant reduction of HR by −9 bpm compared to the initial HR before landiolol hydrochloride injection.

**Table 1:** Heart rate before landiolol hydrochloride administration (HR_PRE_), heart rate after last partial landiolol hydrochloride injection (HR_1–6_), heart rate during cardiac CT (HR_CT_) and heart rate after cardiac CT (HR_POST_), as well as blood pressure (BP_SYST_, BP_DIAST)_ before and after cardiac CT (*n* = 37)

	Median	IQR	Range
HR_PRE_	76 bpm	16 bpm	65–111 bpm
HR_1–6_	60 bpm	6 bpm	55–77 bpm
HR_CT_	63 bpm	12 bpm	48–113 bpm
HR_POST_	71 bpm	12 bpm	47–117 bpm
BP_SYST_PRE_	131 mmHg	26 mmHg	110–179 mmHg
BP_DIAST_PRE_	73 mmHg	15 mmHg	56–96 mmHg
BP_SYST_POST_	119 mmHg	21 mmHg	72–164 mmHg
BP_DIAST_POST_	64 mmHg	10 mmHg	48–86 mmHg

Glyceroltrinitrat was administered in 32/37 patients

**Table 2:** Change in heart rate (HR) and blood pressure (BP_SYST_ and BP_DIAST_) after landiolol hydrochloride injection (*n* = 37)

Difference	Mean ± SD	Lower/upper 95% CI	*p*-value
Total			
HR_1–6_ − HR_PRE_	−14 ± 8 bpm	−17/−11 bpm	< 0.001
HR_CT_ − HR_PRE_	−11 ± 9 bpm	−14/−7 bpm	< 0.001
HR_CT_ − HR_1–6_	3 ± 8 bpm	0/6 bpm	0.027
HR_POST_ − HR_CT_	6 ± 6 bpm	4/8 bpm	< 0.001
Oral beta-blocker			
HR_CT_ − HR_PRE_	−6 ± 5 bpm	−8/−3 bpm	0.005
No oral beta-blocker			
HR_CT_ − HR_PRE_	−14 ± 10 bpm	−18/−10 bpm	< 0.001
Nitroglycerine			
BP_SYST_POST_ − BP_SYST_PRE_	−14 ± 17 mmHg	−20/−8 mmHg	< 0.001
BP_DIAST_POST_ − BP_DIAST_PRE_	−9 ± 8 mmHg	−12/−6 mmHg	< 0.001
No nitroglycerine			
BP_SYST_POST_ − BP_SYST_PRE_	9 ± 15 mmHg	−10/28 mmHg	0.225
BP_DIAST_POST_ − BP_DIAST_PRE_	5 ± 9 mmHg	−6/17 mmHg	0.225

Glyceroltrinitrat was additionally administered in 32/37 patients. Oral Beta-blocker premedication was administered in 14/37 patients

*HR*_*PRE*_ heart rate before landiolol hydrochloride injection, *HR*_*1–6*_ minimal heart rate during partial landiolol hydrochloride injections, *HR*_*CT*_ heart rate during cardiac CT scan, *HR*_*POST*_ heart rate after cardiac CT, *BP*_*SYST_PRE*_ */ BP*_*DIAST_PRE*_ systolic / diastolic blood pressure before landiolol hydrochloride administration, *BP*_*SYST_POST*_ */ BP*_*DIAST_POST*_ systolic / diastolic blood pressure after cardiac CT

During fractional landiolol hydrochloride administration, a HR ≤ 60 bpm was achieved in 22 patients (60%) and a HR of ≥ 60 bpm, but ≤ 65 bpm was achieved in 6 patients.

During CCTA, a HR ≤ 60 bpm occurred in 13 patients (35%) and a HR of ≥ 60 bpm, but ≤ 65 occurred in 12 patients. So overall, during fractional landiolol hydrochloride administration, a HR of ≤ 65 bpm was achieved in 28 patients (76%) and during CCTA, a HR of ≤ 65 bpm was achieved in 25 patients (68%).

Although a statistically significant smaller decrease of HR after landiolol hydrochloride injection occurred in patients with oral beta-blocker premedication than in patients without prior oral medication, the difference between the HR during CCTA and the initial HR was also statistically significant in this subgroup (Table 2).

In patients who additionally received glyceroltrinitrat, statistically significant lower blood pressure was measured after the CT examination compared to the initial blood pressure. On the contrary, no statistically significant change in blood pressure was seen in patients with landiolol hydrochloride medication only (Table 2).

**Fig. 2:**
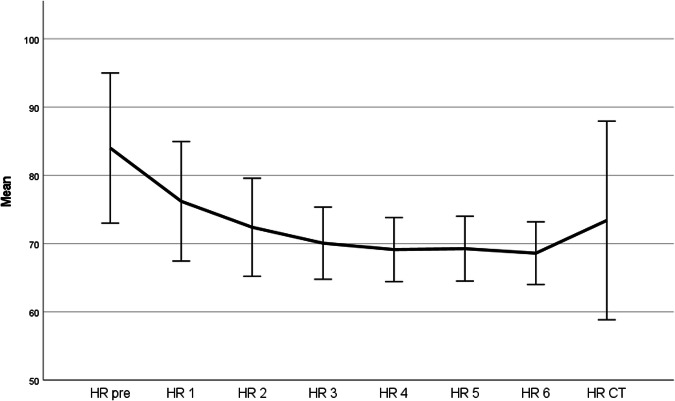
Mean HR and standard deviation after stepwise application of landiolol hydrochloride and during cardiac CT in patients who received the full dose of 60 mg in six injections (15 patients)

In patients who received the full dose (60 mg) of landiolol hydrochloride according to our study protocol (*n* = 15), each partial injection up to the fourth injection led to a statistically significant decrease in the HR compared to the HR after the previous injection. The fifth and sixth injections did not result in a further statistically significant decrease of the HR (Table 3). Figure 2 visualizes the course of the mean HR after the application of each partial dose. A clear reduction of the HR after injection of the first three doses of landiolol hydrochloride was observed. A less distinct, yet significant decrease of the HR after the fourth injection was achieved. However, after the fifth partial injection of landiolol hydrochloride no significant change of the HR was detectable.

**Table 3:** Heart rate before landiolol hydrochloride administration (HR_PRE_) and after each partial injections (HR_1_–HR_6_) in individuals who received all six injections of landiolol hydrochloride (*n* = 15)

	Median	IQR	Range
HR_PRE_	82 bpm	13 bpm	68–111 bpm
HR_1_	75 bpm*	17 bpm	65–95 bpm
HR_2_	70 bpm*	14 bpm	64–86 bpm
HR_3_	68 bpm*	10 bpm	64–80 bpm
HR_4_	68 bpm*	10 bpm	64–77 bpm
HR_5_	68 bpm	9 bpm	64–77 bpm
HR_6_	68 bpm	9 bpm	64–77 bpm
HR_CT_	71 bpm	16 bpm	57–113 bpm

* Statistically significant difference compared to previous HR (*p* ≤ 0.001)

HR decrease following landiolol hydrochloride injection was not influenced by sex (*p* = 0.258), body height (*p* = 0.254), body weight (*p* = 0.285) or body mass index (*p* = 0.180), indicating that the injection schema used in this study compensated for a possible influence of these allometric parameters on landiolol hydrochloride effectiveness.

## Discussion

Our study showed that a statistically significant decrease in the HR can be achieved in CCTA using serial bolus injections of landiolol hydrochloride, which is a beta1-selective antagonist with rapid onset and short duration. Although the decrease of HR was higher in patients without premedication with oral beta-blocker, a significant decrease in HR was also observed in patients with oral beta-blocker premedication. Therefore, landiolol hydrochloride may also be appropriate as an add-on medication in patients who are undergoing a CCTA scan and have a HR > 60–65 bpm despite oral beta-blocker premedication.

During fractional landiolol hydrochloride adinistration, we observed a higher frequency of reaching the target HR compared to the frequency of reaching the target HR during CCTA. This can be explained by the very short duration of action of landiolol hydrochloride. Performing a cardiac CT includes several steps, such as obtaining a topogram, performing a native CT for calcium scoring, and applying a test bolus for transit time determination before the CCTA can be performed. This process takes a few minutes, during which the effect of landiolol hydrochloride may already be diminished. Unfortunately, we do not have records on the exact duration of landiolol hydrochloride injection and CCTA scan per patient. However, a well-trained team of radiology technologists can minimize this time effect.

It has been shown that a bolus injection of landiolol at 0.125 mg/kg in CCTA decreased the HR by −12 bpm at 3–5 min after the injection [12]. In another study, a reduction of HR by −9 bpm was observed following landiolol injection at a bolus dose of 0.125 mg/kg [9]. Hirano et al, who assessed the reductive effects of different doses of landiolol on HR during CCTA, reported that compared with a bolus injection of 0.125 mg/kg, increased doses of 0.25 mg/kg and 0.50 mg/kg resulted in a greater reduction of HR than that by 0.125 mg/kg injection. HR reduction was 16 ± 7 bpm at 0.125 mg/kg, 17 ± 8 mg/kg at 0.25 mg/kg and 22 ± 6 bpm at 0.5 mg/kg. The target HR of 65 bpm was achieved in 59%, 61%, and 63% of patients in groups of 0.125, 0.25, and 0.50 mg/kg injections, respectively [10]. Different patient populations (Asian versus Caucasian) and different approach of landiolol hydrochloride injection (fixed dose per kg versus variable dose) may explain the differences in HR reduction between those studies and our study. However, our research approach was to find a simple, straightforward way of using landiolol hydrochloride to reduce the HR of patients for CCTA using as little amount of the drug as possible and without consideration of allometric parameters. This approach is supported by the study finding that HR decrease following landiolol hydrochloride injection was not influenced by sex, body height, body weight or body mass index.

Due to the rapid effect of landiolol hydrochloride, the change in the HR of patients can be directly observed on the CT scanner. For this reason, the stepwise injection technique, as provided in this study, is a considerable approach of application of landiolol hydrochloride in clinical routine. By using a maximum of 60 mg, we injected serial bolus of landiolol hydrochloride until the target HR of ≤ 60 bpm was reached or the maximum dose was applied. Each partial injection up to the fourth injection led to a significant decrease in the HR compared to the HR after the previous injection, whereas the fifth and sixth injections did not result in a further decrease. This suggests that for CCTA premedication, the maximum dose of landiolol hydrochloride may be limited to 40 mg. An explanation may point to a ceiling effect of landiolol hydrochloride. However, to our knowledge, there is so far no ceiling effect of landiolol hydrochloride described in literature. Another explanation would be justified by the short half-life, as the effect of the first dose could already be diminished when several partial doses are applied. However, this finding may be limited to patients with similar allometric parameters to patients included in our study.

It has been reported that the use of landiolol has no major effects on blood pressure making it a convenient drug to prepare for CCTA [11–15]. This is supported by our study, as a statistically significant decrease in blood pressure only occurred in patients who received glyceroltrinitrat in addition to landiolol hydrochloride. The alterations in blood pressure before and after CT examination shown in Table 1 are primarily explainable due to the fact that most patients in this study group additionally received glyceroltrinitrat. In patients who received landiolol hydrochloride only, no statistically significant change in blood pressure was observed (Table 2). The minor effect on blood pressure may make landiolol more suitable compared to esmolol as premedication for CCTA. Compared to metoprolol, esmolol seems to cause a higher rate of transient hypotension (systolic BP < 100 mmHg) in patients right after the CT scan [16]. Landiolol hydrochloride seems to have a more prominent and pronounced bradycardic effect in relation to its blood pressure-lowering effect [8]. The usage of landiolol hydrochloride is very convenient for patients undergoing CCTA due to its very short duration of action. Table 4 provides a comparison in onset, duration of action and pharmacokinetic properties between landiolol and esmolol and other beta-blockers [3, 4, 17, 18]. Patients usually do not have to be monitored after the CT scan because the effect of landiolol hydrochloride wears off or is already over at the time the patients leave the CT table. In addition, there are no limitations for the patients in their daily routine in the remaining day after the examination. Listed contraindications for the use of landiolol hydrochloride are similar to those of other beta-blocker such as metoprolol or esmolol. According to the manufacturer’s information, contraindications for the use of landiolol hydrochloride are allergic reactions to landiolol or its additives, bradycardia (< 50 bpm), sick-sinus syndrome, high-grade AV-block, cardiogenic shock, low blood pressure, severe heart failure, pulmonary hypertension, severe hypotension, pheochromocytoma, severe asthma, severe metabolic acidosis. However, it is worth mentioning that, to our knowledge, there are no data on the usage and safety of landiolol hydrochloride in children. Some studies have already reviewed the safety of beta-blockers, including landiolol hydrochloride [12, 19]. The studies included patients with premedication such as angiotensin II receptor blocker /angiotensin-converting enzyme inhibitor (ACEI), beta-blockers, and calcium channel blockers. These studies concluded that a bolus injection of landiolol reduced HR without any severe adverse effects. Kokubo et al claim that the administration of beta-blockers immediately before CCTA affects HR but not EF and that premedication with beta-blockers can be safely used for patients who undergo CCTA [19]. In our study, no serious adverse events occurred after the administration of landiolol, even though a premedication with other beta-blockers or additional application of glyceroltrinitrat. The factory-selling price of landiolol hydrochloride should be kept in mind. In the authors’ country, the cost of one ampulla of landiolol hydrochloride (Rapibloc®) 20 mg/2 mL is about four and a half times the amount of one ampulla of esmolol hydrochloride (Esmolol®) 100 mg/10 mL and twenty times the cost of metoprolol (Beloc®). The costs may vary in different countries; nonetheless, landiolol hydrochloride seems to be rather expensive compared to commonly used beta-blockers such as esmolol and metoprolol. This may be a sticking point for institutions to establish the usage of landiolol hydrochloride as a premedication for CCTA.

**Table 4:** Comparison of landiolol hydrochloride to common beta-blockers in their onset of action and t1/2

Drug	Mode of administration	Onset of action (min)	Half-life, t1/2 (min)
Landiolol	i.v.	1	3–4 min
Esmolol	i.v.	2	9
Metoprolol	i.v.	5–10 min	120–180
Metoprolol	Oral	30–40	180–420
Bisprolol	Oral	50–70	540–720
Atenolol	Oral	60–90	360–420

One main limitation of our study must be considered, which is the small and heterogeneous patient population including patients with either no or different oral beta-blocker premedication and use of glyceroltrinitrat. In addition, the majority of included patients had normal cardiac ejection fraction in cardiac ultrasound and CAD-RADS 1–3 reports in CCTA. Therefore, the results of our study may be limited to similar patient populations. Further studies on a larger sample size with the ability of subgroup analysis are advisable to confirm our findings.

In conclusion, landiolol hydrochloride enables a reduction of the heart rate for CCTA in patients with and without oral beta-blocker premedication, whereas the use of serial partial doses is a simple and effective approach in clinical routine, without the need for dose adjustment to body weight.

## Data Availability

Data are available from the corresponding author.

## Abbreviations

Bpm: Beats per minute
BP_POST_: Blood pressure after landiolol hydrochloride injection
BP_PRE_: Blood pressure before landiolol hydrochloride injection
CCTA: Coronary artery CT angiography
HR: Heart rate
HR_1–6_: Heart rate after each of six partial doses of landiolol hydrochloride injection
HR_CT_: Heart rate during CT scan
HR_POST_: Heart rate after the CT examination before moving from the CT table
HR_PRE_: Heart rate before landiolol hydrochloride injection
SD: Standard deviation

## References

[1] Stocker TJ, Leipsic J, Chen MY et al (2021) Influence of heart rate on image quality and radiation dose exposure in coronary CT angiography. Radiology 300:701–703 34128722 10.1148/radiol.2021210245

[2] Ablad B, Borg KO, Carlsson E et al (1975) A survey of the pharmacological properties of metoprolol in animals and man. Acta Pharmacol Toxicol 36:7–23 10.1111/j.1600-0773.1975.tb03318.x 1094804

[3] Tsuchiya H, Mizogami M (2013) Characteristic interactivity of landiolol, an ultra-short-acting highly selective β1-blocker, with biomimetic membranes: comparisons with β1-selective esmolol and non-selective propranolol and alprenolol. Front Pharmacol 4:150 24339816 10.3389/fphar.2013.00150 PMC3857573

[4] Murakami M, Furuie H, Matsuguma K, Wanibuchi A, Kikawa S, Irie S (2005) Pharmacokinetics and pharmacodynamics of landiolol hydrochloride, an ultra short-acting β1-selective blocker, in a dose escalation regimen in healthy male volunteers. Drug Metab Pharmacokinet 20:337–344 16272751 10.2133/dmpk.20.337

[5] Nasrollahi-Shirazi S, Sucic S, Yang Q, Freissmuth M, Nanoff C (2016) Comparison of the β-adrenergic receptor antagonists landiolol and esmolol: receptor selectivity, partial agonism, and pharmacochaperoning actions. J Pharmacol Exp Ther 359:73–81 27451411 10.1124/jpet.116.232884

[6] Barbier GH, Shettigar UR, Appunn DO (1995) Clinical rationale for the use of an ultra-short acting beta-blocker: esmolol. Int J Clin Pharmacol Ther 33:212–218 7620691

[7] Matsuishi Y, Mathis BJ, Shimojo N, Kawano S, Inoue Y (2020) Evaluating the therapeutic efficacy and safety of landiolol hydrochloride for management of arrhythmia in critical settings: review of the literature. Vasc Health Risk Manag 16:111–123 32308404 10.2147/VHRM.S210561 PMC7138627

[8] Krumpl G, Ulc I, Trebs M, Kadlecová P, Hodisch J (2017) Bolus application of landiolol and esmolol: comparison of the pharmacokinetic and pharmacodynamic profiles in a healthy Caucasian group. Eur J Clin Pharmacol 73:417–428 28091703 10.1007/s00228-016-2176-0

[9] Nakamura Y, Yamaji K, Saho T et al (2014) A comparison of bolus injection of landiolol versus oral administration of propranolol before cardiac computed tomography. Springerplus 3:93 24634807 10.1186/2193-1801-3-93 PMC3951651

[10] Hirano M, Hara K, Ikari Y et al (2013) Dose-finding study of landiolol hydrochloride: a short-acting β1-blocker for controlling heart rate during coronary computed-tomography angiography in Japan. Adv Ther 30:803–818 24062147 10.1007/s12325-013-0053-0 PMC3824371

[11] Osawa K, Miyoshi T, Sato S et al (2013) Safety and efficacy of a bolus injection of landiolol hydrochloride as a premedication for multidetector-row computed tomography coronary angiography. Circ J 77:146–152 23037522 10.1253/circj.cj-12-0663

[12] Koyoshi R, Shiga Y, Idemoto Y et al (2018) Safety of landiolol hydrochloride as a premedication for producing an appropriate heart rate for multidetector-row computed tomography coronary angiography. J Clin Med Res 10:22–26 29238430 10.14740/jocmr3213w PMC5722041

[13] Hirano M, Yamashina A, Hara K et al (2014) A multicenter, open-label study of an intravenous short-acting β1-adrenergic receptor antagonist landiolol hydrochloride for coronary computed tomography angiography by 16-slice multi-detector computed tomography in Japanese patients with suspected ischemic cardiac disease. Drugs R D 14:185–194 25091378 10.1007/s40268-014-0056-6 PMC4153968

[14] Kido T, Mochizuki T, Hirano M et al (2016) Radiation-dose-lowering effects of landiolol hydrochloride in coronary angiography using computed tomography (DELIGHT)—a prospective multicenter study. Circ J 80:1225–1231 27019983 10.1253/circj.CJ-15-0962

[15] Isobe S, Sato K, Sugiura K et al (2008) Feasibility of intravenous administration of landiolol hydrochloride for multislice computed tomography coronary angiography: initial experience. Circ J 72:1814–1820 18827370 10.1253/circj.cj-08-0336

[16] Maurovich-Horvat P, Károlyi M, Horváth T et al (2015) Esmolol is noninferior to metoprolol in achieving a target heart rate of 65 beats/min in patients referred to coronary CT angiography: a randomized controlled clinical trial. J Cardiovasc Comput Tomogr 9:139–145 25819196 10.1016/j.jcct.2015.02.001

[17] Ågesen FN, Weeke PE, Tfelt-Hansen P, Tfelt-Hansen J (2019) Pharmacokinetic variability of beta-adrenergic blocking agents used in cardiology. Pharmacol Res Perspect 7:e00496 31338197 10.1002/prp2.496 PMC6624454

[18] Baker JG (2005) The selectivity of β-adrenoceptor antagonists at the human β1, β2 and β3 adrenoceptors. Br J Pharmacol 144:317–322 15655528 10.1038/sj.bjp.0706048 PMC1576008

[19] Kokubo R, Hirano M, Tajima Y, Yunaiyama D, Saito K (2022) Effects of β-blocker administration on cardiac function: a coronary computed tomography angiography study. Curr Med Imaging 18:1517–1525 35593335 10.2174/1573405618666220518104929 PMC9903291

